# *Plasmodium yoelii* surface-related antigen (PySRA) modulates the host pro-inflammatory responses via binding to CD68 on macrophage membrane

**DOI:** 10.1128/iai.00113-24

**Published:** 2024-04-16

**Authors:** Xin Feng, Jia-Li Yu, Yi-Fan Sun, Chen-Yan Du, Yao Shen, Lu Zhang, Wei-Zhong Kong, Su Han, Yang Cheng

**Affiliations:** 1 Department of Public Health and Preventive Medicine, Laboratory of Pathogen Infection and Immunity, Wuxi School of Medicine, Jiangnan University, Wuxi, China; 2 Department of Laboratory Medicine, Affiliated Hospital of Jiangnan University, Wuxi, Jiangsu, China; 3 Department of Food Quality and Safety, School of Food Science and Technology, Jiangnan University, Wuxi, China; 4 Department of General Practice, Rongxiang Street Community Health Service Center, Binhu District, Wuxi, China; University of California Davis, Davis, California, USA

**Keywords:** *Plasmodium yoelii*, surface-related antigen, macrophage, inflammatory response, CD68

## Abstract

Malaria, one of the major infectious diseases in the world, is caused by the *Plasmodium* parasite. *Plasmodium* antigens could modulate the inflammatory response by binding to macrophage membrane receptors. As an export protein on the infected erythrocyte membrane, *Plasmodium* surface-related antigen (SRA) participates in the erythrocyte invasion and regulates the immune response of the host. This study found that the F2 segment of *P. yoelii* SRA activated downstream MAPK and NF-κB signaling pathways by binding to CD68 on the surface of the macrophage membrane and regulating the inflammatory response. The anti-PySRA-F2 antibody can protect mice against *P. yoelii*, and the pro-inflammatory responses such as IL-1β, TNF-α, and IL-6 after infection with *P. yoelii* are attenuated. These findings will be helpful for understanding the involvement of the pathogenic mechanism of malaria with the exported protein SRA.

## INTRODUCTION

Malaria is an important mosquito-borne disease that seriously endangers human life and health. In 2021, global reports indicate that nearly 247 million malaria cases caused 619,000 deaths (1). Malaria is a highly complex disease with a variety of pathologic manifestations. In the early stages of infection, most patients showed general symptoms such as fever, sweat, and headache. Meanwhile, the symptoms of cerebral malaria, severe anemia, and respiratory distress occurred with the increase in parasites during the erythrocytic stage, leading to severe malaria (2–4).

The pathogenic mechanisms of malaria mainly include microvascular obstruction, erythrocyte destruction, and an inflammatory response (5, 6). Systemic inflammation is an important feature of malaria, and the innate immune response plays a crucial role in protective immunity and the pathogenic mechanism of *Plasmodium* (7, 8). Importantly, serving as a pro-inflammatory inducer, exported protein on the infected erythrocyte surface, the malarial hemozoin, glycosylphosphatidylinositol, or DNA complexes of parasites could bind host cell surface receptors or pattern recognition receptors, such as Toll-like receptors, regulating host inflammation (2, 9, 10). The *Plasmodium*-host immune cell interaction will activate cell-associated signaling pathways to produce high levels of pro-inflammatory factors, including tumor necrosis factor (TNF) and interferon-γ (IFN-γ). The pro-inflammatory factors could damage endothelial cells and destroy blood vessel walls by mediating the isolation of *plasmodium*, leading to the exudation of vascular contents and the occurrence of local tissue inflammation (11). Additionally, excessive secretion of pro-inflammatory factors will further interfere with erythrocyte production and aggravate host anemia (12, 13).

Meanwhile, the host immune system also promoted the secretion of pro-inflammatory factors such as IL-1β, IL-6, TNF, and IL-12 by transforming effector cells to eliminate *plasmodium*, inhibit parasite development, and secrete transforming-growth factor-β (TGF-β), IL-10, and other balanced anti-inflammatory factors (2, 14, 15).However, the immune response of the host is maladjusted, and the pro-inflammatory response is over-upregulated, which leads to the dysfunction of the host cardiovascular or coagulation system as well as systemic and organ-related diseases. An excessive inflammatory response is closely related to severe malaria and patient death (2, 11, 16). However, the mechanism of the host pro-inflammatory response caused by the *plasmodium* proteins remains poorly understood.

The spleen is an important immune and hematopoietic organ that can selectively filter and remove abnormal, aged, or damaged erythrocytes and pathogens (6). The spleen enhances host antigen-specific responses via phagocytosis of infected erythrocytes and parasite components via antigen-presenting cells (6, 17, 18). Macrophages from the host spleen mediate phagocytic activity and cytokine production during the erythrocytic stage by interacting with parasite antigens on the surface of infected erythrocytes; thus, this process is an essential part of the host innate immune system (19).

*P. falciparum* surface-related antigen (SRA) is located on the surface of merozoites and gametocytes, exported to the surface of infected erythrocyte membranes, and further bound to human erythrocytes (20). Studies have shown that the N-terminal-specific antibody of PfSRA could effectively inhibit the invasion of merozoite into erythrocytes (21). The SRA protein is regarded as a potential candidate antigen for the malaria vaccine because the N-terminal of PfSRA is highly conserved by gene polymorphism analysis (22). In addition, PvSRA could interact with Integrin β1 (ITGB1) on the surface of human splenic fibroblasts (HSFs), participating in the regulation of immune responses (23). However, the functional properties of SRA cannot be verified *in vivo* due to moral and ethical constraints. *P. yoelii* has been widely studied due to its biological similarity to *P. falciparum* and *P. vivax*. As the homologous protein of PfSRA and PvSRA, PySRA has a high degree of homology, indicating the potential role of PySRA in regulating the host immune response. Therefore, a mouse model of *P. yoelii* infection was used to characterize the functional properties of SRA and explore the pathogenic mechanism of *Plasmodium*.

## RESULTS

### Schematic of the primary structure, expression, and purification of PySRA

**Fig 1:**
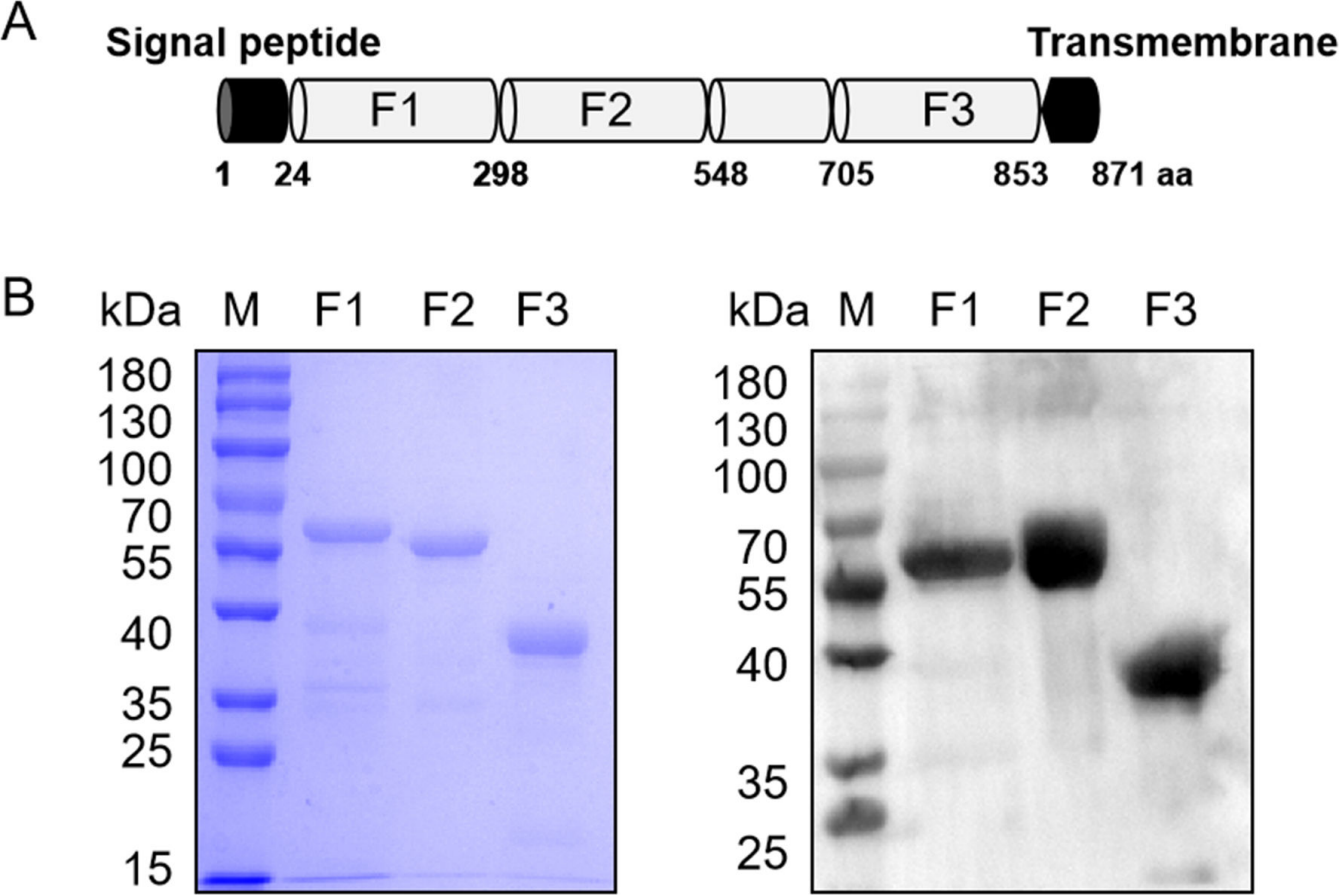
Schematic diagram and polymerase chain reaction (PCR) amplification of PySRA fragments. (**A**) Schematic diagram of PySRA. The PySRA protein contains 871 aa with a predicted signal peptide (1–24 aa) and a transmembrane domain (853–871 aa). PySRA-F1 fragment (25–298 aa), PySRA-F2 fragment (299–548 aa), and PySRA-F3 fragment (705–852 aa) were constructed for expression. (**B**) The recombinant protein purification by Coomassie blue-stained SDS-PAGE gels(right) and SDS-PAGE(left) was verified. Recombinant PySRA-F1 (~56 kDa), PySRA-F2 (~52 kDa), and PySRA-F3 (~38 kDa). M: Protein marker.

Due to the large molecular weight of PySRA protein (encoded by PYYM_1014900) , which consists of 871 amino acids (aa), it is difficult to express in the *Escherichia coli* system. Furthermore, the N-terminal and C-terminal signal peptides and transmembrane region of PySRA may not be correctly recognized and folded by the *E. coli* system. Therefore, during gene amplification, these two structural domains were removed. Based on its structural characteristics and homology comparison with PfSRA fragments (encoded by PF3D7_1431400) (20, 21), three segments of PySRA were selected and successfully expressed. The PySRA protein contains a signal peptide (aa 1–24) and a transmembrane domain (aa 853–871). The codon-optimized *PySRA* gene, which has a flag tag at the 3′ end, was cloned into the pET30a vector for histidine (His)-tagged recombinant protein expression in *E. coli* (Fig. 1A). The results from the SDS–PAGE and Western blot showed that the recombinant PySRA-F1 (aa 24–298), PySRA-F2 (aa 299–548), and PySRA-F3 (aa 705–852) proteins with His-tag were successfully expressed and migrated at approximately 56, 52, and 38 kDa, respectively, under reducing conditions confirmed by SDS-PAGE and immunoblot using an anti-His antibody (Fig. 1B).

### Anti-PySRA-F2 antibody had a protective effect on mice against *P. yoelii* 17XL

**Fig 2:**
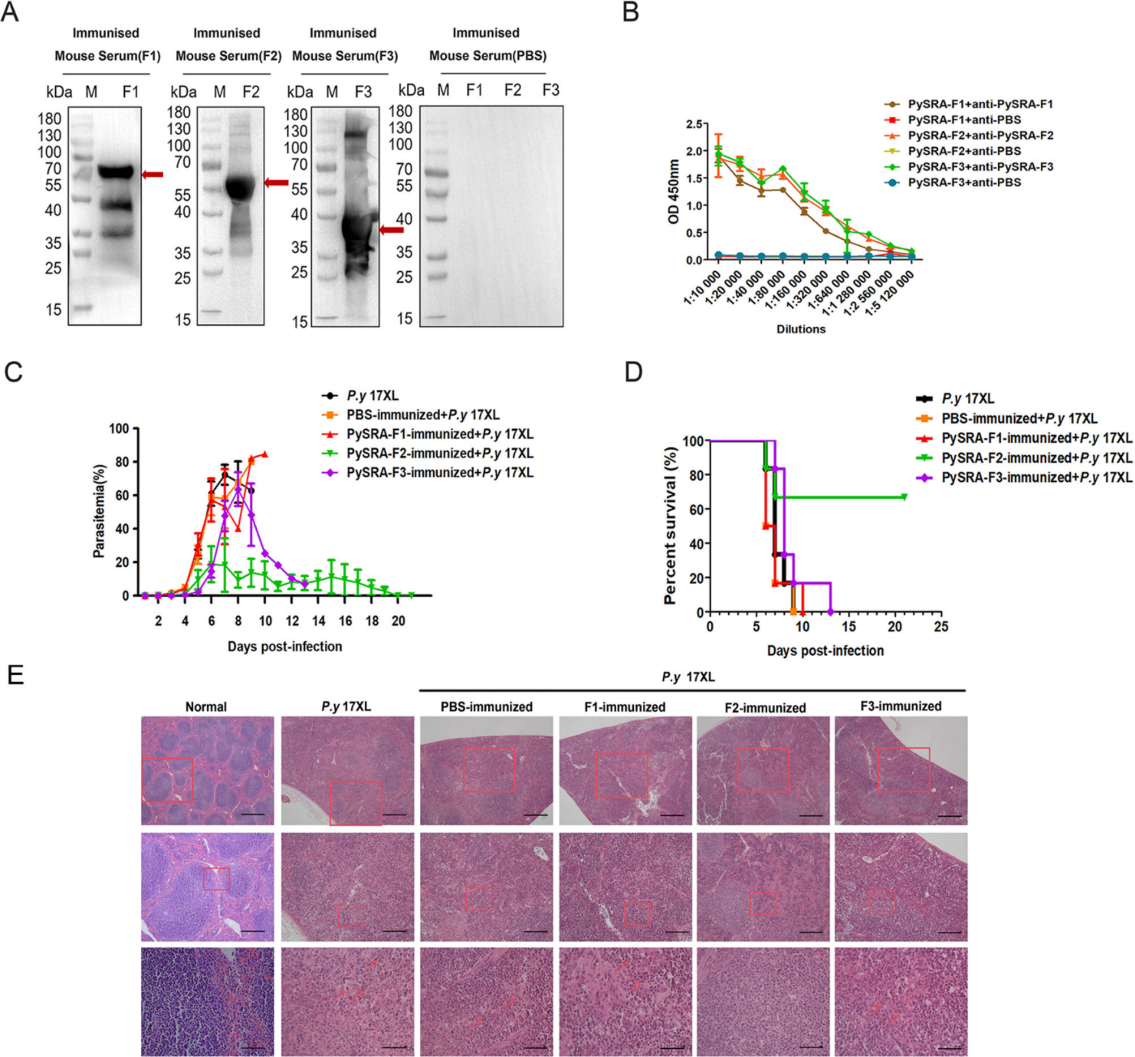
Anti-PySRA-F2 antibody had protective effect on mice infected with *P.y* 17XL. (**A**) The recombinant PySRA could be specifically recognized by the sera of mice immunized with PySRA. M: Protein marker. (**B**) Detection of antibody titer in serum of mice immunized with recombinant PySRA protein. (**C**) The parasitemia of immunized mice infected with *P.y* 17XL. (**D**) The percent survival of immunized mice infected with *P.y* 17XL. (**E**) HE staining results of mice spleen infected with *P.y* 17XL. The red arrow indicates malarial hemozoin (Scale bars: top, 200 µm; mid, 100 µm; bottom, 20 µm).

The corresponding recombinants were recognized using mouse sera (1:1,000) after immunizing mice with the recombinant PySRA fragments (Fig. 2A). The titers of specific IgG in the protein-immunized mice sera were then detected by enzyme-linked immunosorbent assay (ELISA). Detection results revealed that PySRA-F1, PySRA-F2, and PySRA-F3 induced a high immune response in mice with endpoint titers ranging from 1:10,000 to 1:5,120,000 (Fig. 2B), which indicated that the mice model containing anti-PySRA antibody was successfully constructed.

The mice were then infected with *P.y* 17XL on day 7 after immunization. Parasitemia and the health status of the mice were observed daily. The results showed that the parasitemia of mice immunized with PySRA-F2 was at a continuously low level, and undetected parasite growth was observed in the surviving mice on day 21 after infection. The parasitemia of mice in PySRA-F1, PySRA-F3, the PBS immunized group, and the *P.y* 17XL control group continued to rise to approximately 80% (Fig. 2C). In addition, the survival rate of mice in the PySRA-F2 immunized group was significantly improved, reaching 66.67%. Meanwhile, all mice in the four other groups died around 9–13 days after infection (Fig. 2D), and only the PySRA-F2-immunized group gained weight slowly, while the other immunized groups lost weight (Fig. S1A), which proved that the antibody induced by PySRA-F2 in mice had a protective effect on the host. Compared with the uninfected mice, the boundary between the red and white pulps of spleen was unclear and hemozoin was observed (indicated by the red arrow) in *P.y* 17XL-infected mice and the PBS and PySRA-F1 immunized groups; although boundary between the red and white pulps of the spleen of PySRA-F3 immunized groups was able to be seen, a large amount of hemozoin was found. Particularly, the boundary was defined in the mice immunized with the recombinant protein PySRA-F2, indicating that the spleen structure of mice in this group was complete (Fig. 2E; Fig. S2).

### PySRA regulated the host pro-inflammatory response and induced macrophage apoptosis

**Fig 3:**
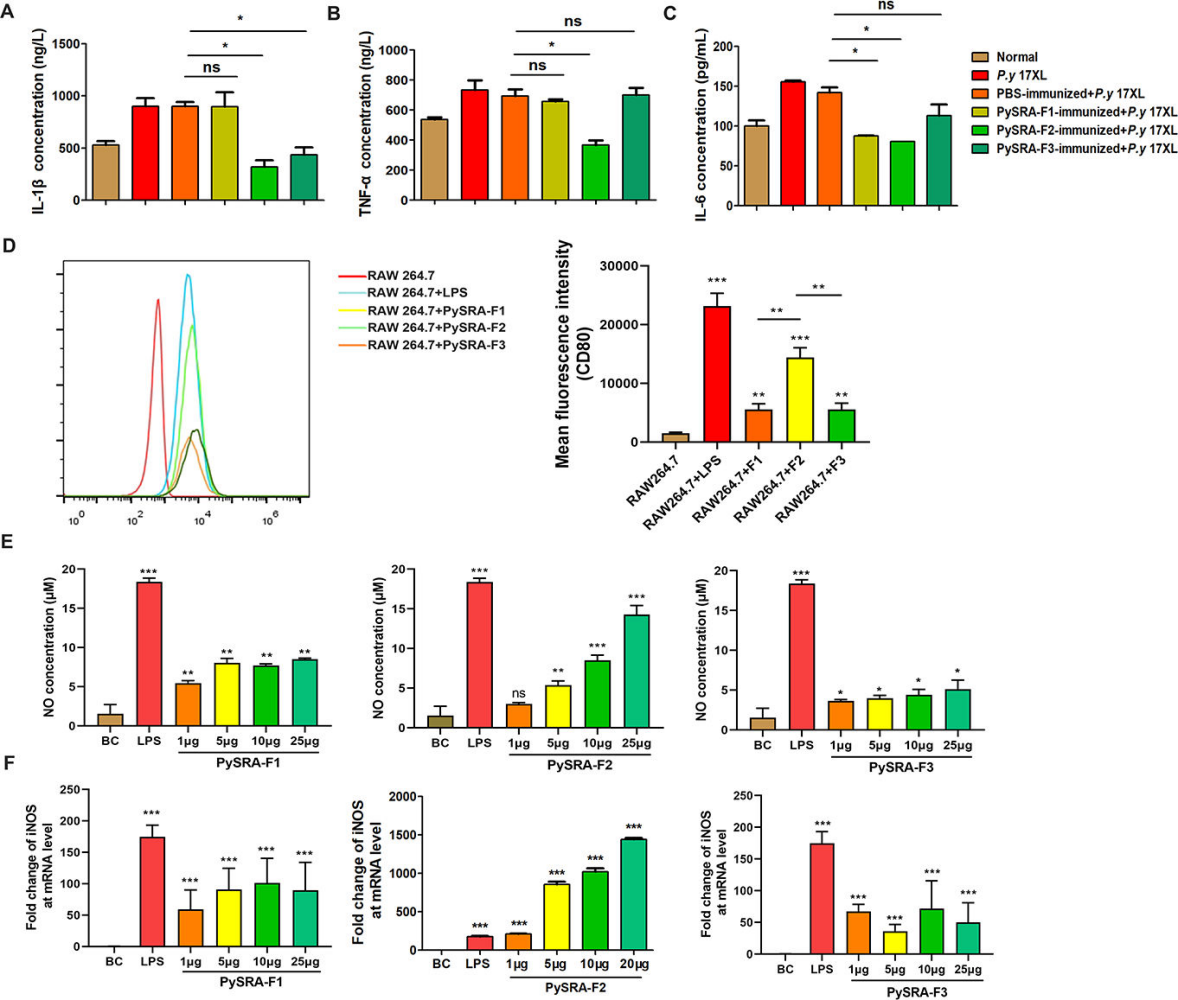
PySRA-F2 regulates macrophage-mediated inflammatory responses. (**A**) The IL-1β pro-inflammatory factor in mice sera was detected by ELISA (*P < 0.05; ns, no significance). (**B**) The TNF-α pro-inflammatory factor in mice sera was detected by ELISA (*P < 0.05; ns, no significance). (**C**) The IL-6 pro-inflammatory factor in mice sera was detected by ELISA (*P < 0.05; ns, no significance). (**D**) PySRA-F2 induced M1 RAW264.7 cells polarization (***P < 0.001). (**E**) NO secretion from RAW264.7 cells was promoted by PySRA-F2 (**P < 0.01; ***P < 0.001; ns, no significance). (**F**) PySRA-F2 promoted the mRNA expression of NO in RAW264.7 cells (***P < 0.001). (**G**) IL-1β transcription levels after PySRA-F2 action on RAW264.7 macrophages (***P < 0.001). (**H**) TNF-α transcription levels after PySRA-F2 action on RAW264.7 macrophages (***P < 0.001). (**I**) IL-6 transcription levels after PySRA-F2 action on RAW264.7 macrophages (***P < 0.001). One-way ANOVA was used for the comparison of multiple groups of samples, and then the SNK test was used for pound-for-pair comparison.

The PySRA-F2 immunized group at the later stage of infection invaded reticulocytes dominantly, while parasites from the four other groups continued to invade mature erythrocytes (Fig. S1B and C). In addition, a wealth of immune cells only appeared in the blood of mice immunized with PySRA-F2 protein rather than the four other groups, suggesting that protective effects in PySRA-F2 immunized mice are likely due to modulation of key host immune responses (Fig. S1B). In view of the inflammatory response as an important part of host immune regulation during *Plasmodium* infection, serums were collected in each group of mice. The results showed that the levels of IL-1β, TNF-α, and IL-6 in the sera of mice infected with *P.y* 17XL were significantly increased, and the level of pro-inflammatory factors was inhibited when the mice obtained anti-PySRA-F2 antibodies (Fig. 3A through C).

Macrophages play an important role in the inflammatory response. Therefore, whether PySRA mediates the mechanism of the inflammatory response by modulating macrophages was further explored. *In vitro* cell experiments were conducted for verification. Results showed that after PySRA-F1, PySRA-F2, and PySRA-F3 treated RAW264.7 cells, the cell morphology was fusiform and extended pseudopodia, while the negative control indicated the absence of activation-related morphological changes. Flow cytometry results showed that the cell-surface marker CD80 was significantly increased in the LPS (positive control), PySRA-F1, PySRA-F2, and PySRA-F3 groups. The average fluorescence intensity was statistically significant compared with the negative control group (*P* < 0.05) (Fig. 3D). However, the PySRA-F2 group was more significant compared to the PySRA-F1 and PySRA-F3 groups (*P* < 0.05). The results indicated that PySRA could activate and promote M1 polarization in RAW264.7 cells. PySRA-F1 and PySRA-F3 promote NO secretion by macrophages, and PySRA-F2 enhances NO secretion by macrophages in a dose-dependent manner. With LPS as the positive control, the PySRA-F1/2/3 group was compared to the blank control (BC) group, and the difference was statistically significant (*P* < 0.05) (Fig. 3E). The mRNA level of iNOS was also significantly increased (*P* < 0.05) (Fig. 3F).

**Fig 4:**
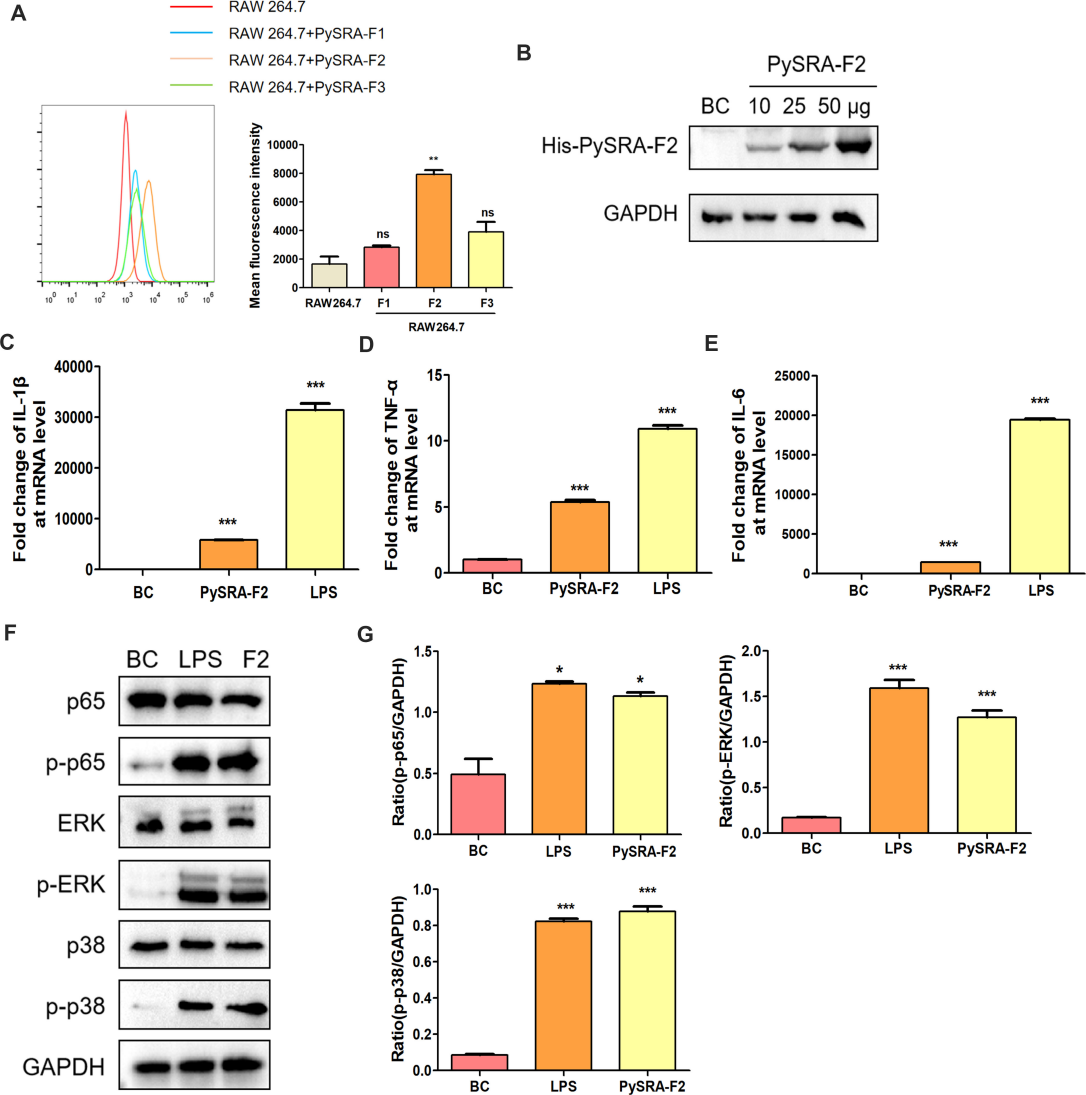
PySRA-F2 binds to RAW264.7 cells. (**A**) The binding ability of PySRA-F2 and RAW264.7 cells by Flow cytometry (**P < 0.01; ns, no significance). (**B**) The binding ability of PySRA-F2 and RAW264.7 cells by Western blot. (**C**) NF-κB and MAPK signaling pathways in RAW264.7 cells were activated by PySRA-F2. M: Protein marker. (**D**) Statistical analysis of p65, ERK, and p38 protein phosphorylation in macrophages after 15 min stimulation (*P < 0.05; ***P < 0.001). One-way ANOVA was used for the comparison of multiple groups of samples, and then the SNK test was used for pound-for-pair comparison.

PySRA is an exported protein and has a theoretical basis for interacting with host cells. Therefore, whether PySRA could bind to macrophages was first analyzed. Flow cytometry results showed that PySRA-F2 could effectively bind to RAW264.7 cells, and the average fluorescence intensity was significantly higher than that of the control group (*P* < 0.05), indicating that the F2 segment is an important part of PySRA in the interaction with RAW264.7 cells (Fig. 4A). Western blot analysis results further proved that PySRA-F2 was bound to RAW264.7 in a dose-dependent manner (Fig. 4D). This suggests to us that PySRA-F2 is the main fragment of PySRA protein that interacts with macrophages and exerts its functions. Further studies have shown that the transcription levels of inflammatory cytokines IL-1β, TNF-α, and IL-6 were significantly increased (*P* < 0.05) (Fig. 4C through E). NF-κB and MAPK are important pathways of pro-inflammatory cytokines secreted by macrophages. The phosphorylation of p65, ERK, and p38 proteins significantly increased based on Western blot analysis (*P* < 0.05) (Fig. 4F and G), which indicated PySRA-F2 could activate the NF-κB and MAPK signaling pathways in RAW264.7 cells.

The effect of PySRA protein on macrophage activity was further detected using the CCK-8 kit. Compared with the LPS group, the viabilities of RAW264.7 cells were significantly decreased in the PySRA-F2 group (*P* < 0.05) in a dose-dependent manner (Fig. S1D). The proportion of macrophage apoptosis gradually increased with the duration of PySRA-F2 treatment via flow cytometry, while that of apoptotic cells was maximized at 24 h of stimulation (*P* < 0.05). The results indicated that the PySRA-F2 protein induced macrophage apoptosis (Fig. S1E).

### PySRA-F2 bound to CD68 on the membrane of macrophages

**Fig 5:**
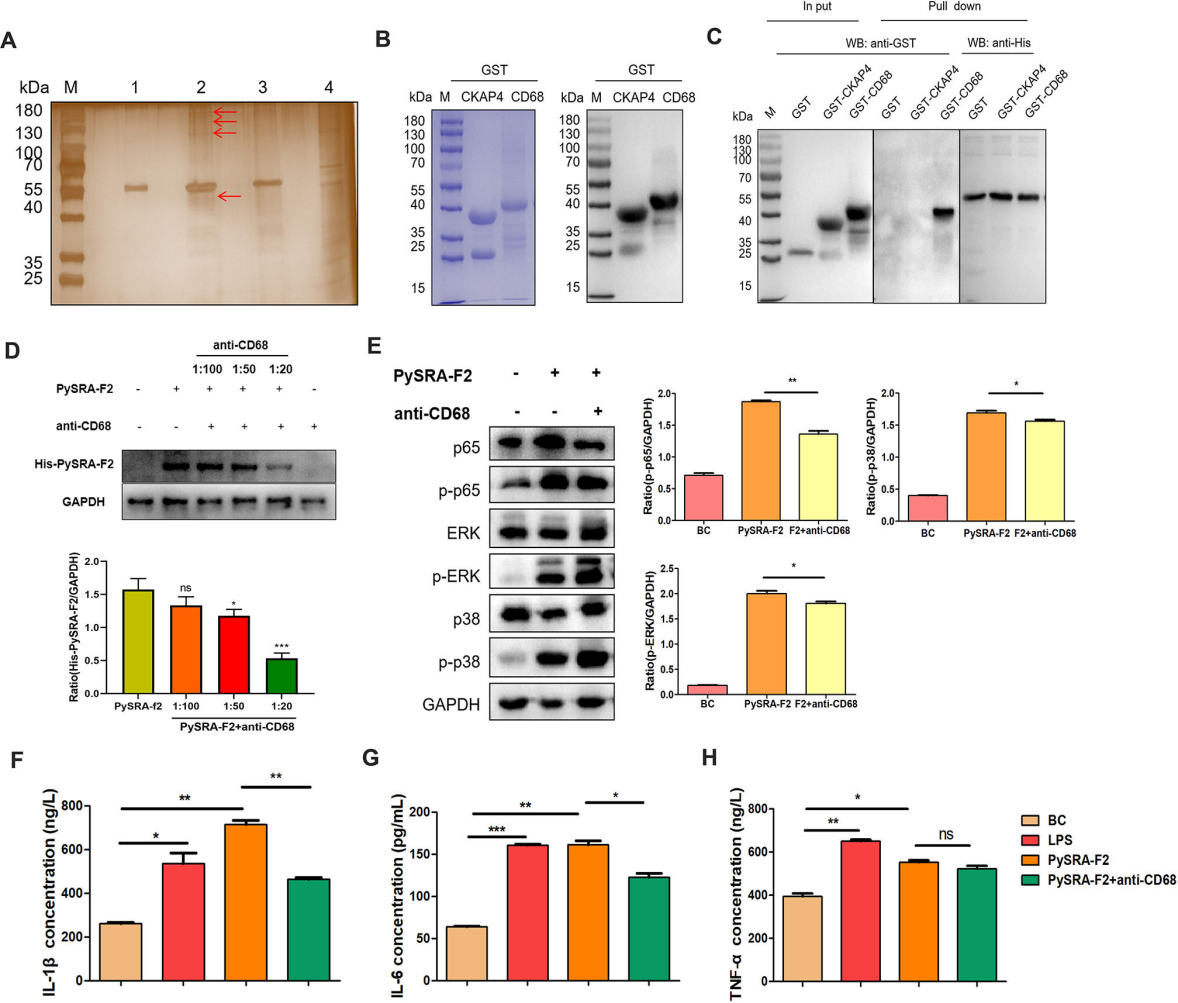
CD68 blockade reduces pro-inflammatory responses. (**A**) To screen the RAW264.7 cell membrane proteins binding to PySRA by Silver staining. 1: Recombinant PySRA-F2 purified protein. 2: The first 200 mM imidazole eluent. 3: The second 200 mM imidazole eluent. 4: RAW264.7 cell membrane proteins. (The red arrows point to bands with differences.) (**B**) The recombinant protein purification by SDS-PAGE was verified. Recombinant protein of CKAP4 (~38 kDa), and CD68 (~41 kDa). M: Protein marker. (**C**) PySRA-F2 binds to CD68 molecule in the macrophage membrane protein. (**D**) The CD68 antibody attenuated the binding of PySRA to macrophages (**P < 0.01). (**E**) The CD68 antibody attenuated the activization of MAPK and NF-κB signaling pathways (*P < 0.05; **P < 0.01). (**F**) Anti-CD68 antibody attenuated the level of IL-1β secretion of RAW264.7 cells regulated by PySRA-F2 (*P < 0.05; **P < 0.01). (**G**) Anti-CD68 antibody attenuated the level of IL-6 secretion of RAW264.7 cells regulated by PySRA-F2 (*P < 0.05; **P < 0.01; ***P < 0.001). (**H**) Anti-CD68 antibody attenuated the level of TNF-α secretion of RAW264.7 cells regulated by PySRA-F2 (*P < 0.05; **P < 0.01; ns, no significance). One-way ANOVA was used for the comparison of multiple groups of samples, and then the SNK test was used for pound-for-pair comparison.

The pulldown assay and silver staining were then used to screen out macrophage cell membrane receptors that could bind to PySRA. PySRA-F2 recombinant protein with His label was fixed to Ni-NTA resin, and the binding protein then captured RAW264.7 cell membrane protein. Macrophage cell membrane proteins were extracted for SDS–PAGE and silver staining. Compared with PySRA-F2 pure protein and RAW264.7 cell membrane protein, four distinct bands were screened out in lane 2 of F2, thereby stimulating the macrophages (Fig. 5A). The differential protein bands were excised from the gels for in-gel digestion and LC-MS detection. Peptides that were identified include CD68, CKAP4, Ras GTPase-activating-like protein IQGAP1, and sodium/potassium-transporting ATPase subunit alpha-1 (Table 1). Among them, CD68 and CKAP4 were the two proteins with the highest number of unique peptides identified.

**TABLE 1:** Identification of RAW264.7 membrane proteins binding to PySRA-F2in digested bands excised from the silver-stained gel by LC-MS

No.	Protein	Accession number	Coverage (%)	Mol.wt（kDa）	Unique peptides
1	Macrosialin（CD68）	P31996	6	34.8	7
2	Cytoskeleton-associated protein 4 (CKAP4)	Q8BMK4	7	63.7	3
3	Ras GTPase-activating-like protein IQGAP1	Q9JKF1	5	188.6	2
4	Sodium/potassium-transporting ATPase subunit alpha-1	Q8VDN2	3	112.9	2

CKAP4 and CD68 were cloned, expressed, and purified with a GST tag (Fig. 5B). Strong interactions could be observed between GST-CD68 and PySRA through the GST pulldown assay, while GST and GST CKAP4 were not bound to PySRA. These data indicated that CD68 is the core region of RAW264.7 bound to PySRA (Fig. 5C).

Serum was obtained and purified to acquire polyclonal rabbit antibodies after immunizing rabbits with CD68 protein. Western blot results showed that CD68 polyclonal antibody could specifically recognize the target protein of CD68, and no serum reactivity was observed before immunization, suggesting that CD68 protein induced specific antibody production in the reaction of animals and formed specific antibodies (Fig. S1F). The rPySRA-F2 protein was added and incubated after the RAW264.7 cells were treated with different concentrations of anti-CD68 antibodies. The results showed that the binding of rPySRA-F2 to macrophages was weakened when RAW264.7 cells were treated with a 1:100 dilution of anti-CD68 antibody and displayed minimum efficiency at a 1:20 dilution (Fig. 5D). RAW264.7 cells were treated with anti-CD68 antibody diluted at 1:20 and incubated with rPySRA-F2 protein for 15 min. Western blot results showed that after CD68 antibody weakened the binding of PySRA to macrophages, the phosphorylation levels of p38, ERK, and p65 proteins were significantly downregulated in the MAPK and NF-κB signaling pathways (*P* < 0.05) (Fig. 5E). In addition, compared with group BC, the PySRA-F2 inducing the secretion of IL-1β, IL-6, and TNF-α in macrophages was significantly upregulated (*P* < 0.05). The secretion of IL-1β and IL-6 was attenuated when anti-CD68 antibodies blocked the RAW264.7 macrophage surface receptor (Fig. 5F through H). This finding suggested that PySRA enhanced the inflammatory response of macrophages through the CD68 molecule.

### *PySRA* and *PySRA-f2* could not be knocked out

The construction of the knockout strain was planned to further determine the role of PySRA in the murine malaria model *in vivo*. The schematic of the *PySRA* gene knockout plasmid was shown in Fig. S2A. The left and right homologous arms were designed to be left and right homologous on the pYCm vector, respectively, and the sequence between the arm restriction sites was used as the in-sequence to replace the *PySRA* or *PySRA*-F2 target gene sequence. The right and left homologous arms of the *PySRA* or *PySRA*-F2 knockout plasmid were successfully amplified, and gene knockout plasmids were effectively constructed (Fig. S3B). The successfully constructed knockout plasmids were electrocuted to the purified *P.y* 17XL schizont phase and then infected with new ICR mice. Afterward, these mice were treated with pyrimethamine after initial observation of *Plasmodium*. However, no resistant strains grew in the mice with the addition of pyrimethamine, confirming that the *PySRA* full-length and *PySRA*-F2 genes could not be knocked out (Fig. S3C).

## DISCUSSION

The interaction between the *plasmodium* and the host cells leads to dysregulation of the host immune response (2, 11). The inflammatory response is over-upregulated, which, in turn, leads to dysfunction of the host cardiovascular or clotting system and systemic and organ dysfunction; excessive inflammatory response is also closely associated with severe malaria and patient death (10, 17, 24). This study found that PySRA could bind to macrophages through the CD68 molecule, activating downstream MAPK and NF-κB signaling pathways and thereby promoting the release of pro-inflammatory factors and aggravating *plasmodium* infection (Fig. 2). It is also possible that the anti-PYSRA antibody limits Plasmodium invasion of erythrocytes and reduces parasitemia, but the validation of the binding site of SRA protein to the erythrocyte membrane and further animal experiments are needed to verify the possibility of this conjecture, and from the results of the current experiments, it is possible that the anti-PYSRA antibody prevents PYsra from binding to CD68, which reduces the inflammation and alters the outcome of infection in the mice. These results confirm that PySRA is involved in the regulation of host inflammatory responses, which provides a new basis for SRA protein as a candidate antigen for malaria vaccines.

Three PySRA fragments were expressed in accordance with their structural characteristics and homology comparison with PfSRA. The corresponding recombinant proteins were recognized in the serum of mice after their immunization with the recombinant protein, indicating that specific antibodies were obtained in mice. In the PySRA-F2 immunized group, mice had a low level of parasitemia, and their survival rate was significantly improved. The spleen has three structures: red pulp, white pulp, and marginal region (18). The white pulp comprises immune cells, and the red pulp accounts for 75% of the spleen volume containing fibroblasts and macrophages, which is a microenvironment suitable for accomplishing multiple functions (20–22). The spleen structure of PySRA-F2 immunized mice was intact, which was helpful for clearing the infected erythrocytes and activating the host immune response (Fig. 2). The infected erythrocytes were mechanically isolated, whereas immune cells in the spleen clear the parasite via phagocytosis and upregulation of the antigen-processing machinery and molecules such as MHC class II, CD80 (18, 25, 26). Reports indicated that the clearance rate of infected erythrocytes was significantly reduced in *P. falciparum* malaria patients and rodents after splenectomy (27–30). Compared with patients with normal splenic function, those with splenectomy have a slower rate of malaria parasite clearance and a longer duration of infected erythrocytes in the blood circulation after drug treatment (27–29). The number of immune cells in the blood of PySRA-F2 immunized mice increased, and the pro-inflammatory response was alleviated. These results indicated the protective effect of the anti-PySRA-F2 antibody on mice infected with *P. yoelii* (Fig. 3).

The innate immune system of the host induces macrophages after infection with *Plasmodium* to secrete pro-inflammatory agents and chemokines to eliminate parasites. However, parasite*–*host interactions lead to immune response dysregulation and excessive inflammatory responses, which, in turn, promote the rapid growth of *Plasmodium* (31, 32). In severe malaria patients, the inflammatory response is unbalanced, demonstrating hypersecretion of pro-inflammatory factors and decreased levels of anti-inflammatory factors (11). Consequently, a systemic inflammatory response is triggered, leading to symptoms such as anemia, metabolic acidosis, and multiple organ failure (6). Therefore, inflammation is the core event of malaria-related syndrome, and identifying its mechanism is helpful for the diagnosis and treatment of malaria. Macrophages are important sources of pro-inflammatory factors in the inflammatory response, and *Plasmodium* interacts with macrophages and initiates cytogenesis. Therefore, elucidation of host protective and pathological inflammatory response balance factors can help design targeted regulation of macrophages and therapeutic approaches to cellular function (19). This study confirmed that PySRA-F2 can promote macrophages to the pro-inflammatory M1-type pole to facilitate their activation, which induces the production of pro-inflammatory cytokines. The testing level results for pro-inflammatory factors revealed that the PySRA-F2 protein upregulated the transcription of iNOS, IL-1β, IL-6, and TNF-α cytokines in macrophages. This upregulation confirmed that PySRA-F2 promotes macrophage-mediated inflammatory responses.

The MAPK and NF-κB pathways play important roles in regulating the inflammatory response (33), especially for the secretion of pro-inflammatory factors in macrophages (11). Macrophages are involved in regulating inflammation during *Plasmodium* infection by interacting with infected erythrocytes. The obtained data showed that the incubation of RAW 264.7 macrophages with PySRA-F2 resulted in a reduction of macrophages, while the NF-κB and MAPK signaling pathways were activated with the negative PySRA-F2 concentration. This phenomenon leads to an imbalance in the immune function of hosts. The imbalance of the inflammatory response was reported in severe malaria patients (11), which leads to systemic inflammation and tissue damage in the host (34).

The exported protein PySRA provides the theoretical basis for *Plasmodium*–host cell interaction. After co-incubation with RAW264.7 macrophages, only PySRA-F2 was found to bind to macrophages, and the binding efficiency displayed a dose-dependent relationship with the rPySRA-F2 protein (Fig. 4). CD68, which is one of the glycosylated membrane proteins, is highly expressed in mouse macrophages and regulates macrophage phagocytosis, including intracellular lysosome metabolism as well as cell–cell and cell–pathogen interactions (35, 36). Results also showed that the P38 peptide of sporozoite binds to the CD68 receptor in Kupffer cells, and the invasion rate was significantly reduced in CD68(−/−) mice (36). After blocking the macrophage surface receptor with the CD68 antibody, the PySRA effectively weakened the binding of macrophages and downregulated the phosphorylation levels of ERK, p38, and p65 proteins in the MAPK and NF-κB pathways, as well as the levels of IL-1β and IL-6 significantly reduced (Fig. 5). The PySRA protein is assumed to interact with macrophages through the CD68 receptor and further activates MAPK and NF-κB pathways, which are involved in regulating macrophage-mediated inflammatory responses.

The proposed CRISPR/Cas9 gene editing technique was used to screen the *PySRA* knockout strain *in vivo* to further explore the specific regulation mechanism of PySRA on the host inflammatory response. The CRISPR/Cas9 technique comprises Cas9 endonuclease and guides RNA (gRNA), which helps Cas9 achieve specific recognition and cleavage of target sequence sites (37–39). In this study, no resistant strains were found after electroconversion and pyrimethamine screening. Several repeated sgRNA experiments were designed. However, the full length and each fragment of the *PySRA* gene could not be knocked out, which indicated that *PySRA* was essential for parasite growth and, thus could not be knocked out (Fig. S2).

Overall, the study identified that the PySRA-F2 segment is a crucial functional segment of PySRA. PySRA-F2 may activate MAPK and NF-κB signaling pathways by binding to the CD68 molecule on the surface of the macrophage cell membrane and regulating pro-inflammatory responses. The PySRA-F2 antibody has a protective effect on mice infected with *P. yoelii* 17XL. The RAW264.7 macrophages used in this experiment were from a single cell line, and further validation is needed for primary cells, and the functional properties of SRA from human *plasmodium falciparum* and *vivax* could be further verified.

## MATERIALS AND METHODS

### Expression and purification of recombinant PySRA

The nucleotide sequence encoding the full-length of *pysra* was obtained from the PlasmoDB website (PYYM_1014900), synthesized by TianLin Biotech (Wuxi) with codon optimization for expression in *E. coli* system, and cloned into the pET30a vector. The *pysra*-F1, *pysra*-F2, and *pysra*-F3 fragments were amplified by polymerase chain reaction (PCR) from the full-length gene, and the PCR products were purified by 2% agarose gel electrophoresis and cloned into a pET32a vector (Primer sequences in Table S1). Recombinant plasmids of *pysra* were transformed into *E. coli* BL21 (DE3) pLysS cells (TransGen Biotech) and then grown in Luria Bertani broth containing ampicillin (50 µg/mL) at 37°C for 12 h. The culture was induced by 0.1 mM isopropyl β-d-1-thiogalactopyranoside (IPTG; TransGen Biotech) when the optical density at 600 nm (OD600) reached 0.4–0.6 and was allowed to grow for another 8 h at 37°C. Finally, the protein was purified by TianLin Biotech (Wuxi).

### Immunization and infection of mice

Thirty 6-week-old female BALB/c mice (Cavens) were randomly divided into five groups (six mice per group). Mice in experimental groups were immunized with PySRA-F1, PySRA-F2, and PySRA-F3 recombinant protein, respectively. Then, 50 µg of PySRA protein in PBS (Total 100 µL) was emulsified with 100 µL complete Freund’s adjuvant (CFA; Sigma) at a volume ratio of 1:1 with a total volume of 200 µL in the prime boost. The mixture was intraperitoneally injected into the mice. Additionally, 50 µg of PySRA protein in PBS (Total 100 µL) was emulsified with 100 µL incomplete Freund’s adjuvant (IFA; Sigma) and was administered on days 21 and 42 post-immunization to boost the immunization. Control mice were immunized with CFA/IFA in emulsification with PBS. On the 7th day after the last immunization, serum was prepared from 50 µL of tail blood from each immunized mouse. And the tail blood of mice infected with *P. yoelii* 17XL was collected in PBS, and the tail blood was used to prepare blood smear. After Jimsa staining, the parasitemia was observed and calculated under the microscope. Red cell suspension was prepared according to the number of red blood cells and parasitemia. After infection, the status of mice was observed every day to analyze the survival rate of mice. At the same time, tail blood of mice was taken to prepare blood smear for Jimsa staining, and parasitemia of mice was observed and calculated under the microscope. *P. yoelii* 17XL were maintained in our laboratory.

To ensure viability of the parasites, we thawed a frozen aliquot and inected it intraperitoneally into a transfer Balb/c mice (Cavens), before using them for infection of experimental mice. Experimental mice were infected with 1 × 10^5^ *P. yoelii*-parasitized RBCs by intraperitoneally(i.p.) injection on day 0. On the 5th day post-infection with *P. yoelii*, the mice were anesthetized with 20% ethyl carbamate (Macklin, China) solution by intraperitoneal injection. Blood was collected from the eyeball of the mice and transferred to 1.5  mL microcentrifuge tube and at room temperature for a duration of 30 min, followed by centrifugation at 2,000 × *g* for 10 min. The upper layer of serum was then carefully aspirated and transferred into a new 1.5-mL microcentrifuge tube, properly labeled, and stored at a temperature of −80°C in a refrigerator. Following euthanasia, the spleen was harvested from mice and placed into 4% paraformaldehyde (Meilunbio, China) for over 24 h.

To generate rabbit anti-PySRA-F1/F2/F3 antisera and rabbit anti-CD68 antisera, one female New Zealand white rabbit (2 months old) was injected intramuscularly with 500 µg of PySRA-F1/F2/F3 protein or CD68 protein mixed in Freund’s complete adjuvant, followed by two boost injections with 500 µg of PySRA-F1/F2/F3 or CD68 mixed in Freund’s incomplete adjuvant at 4 weeks intervals. The prime and the final boosts were given by intramuscular injection and intravenous injection, respectively. Antisera was collected 2 weeks after the final boost. Among the above experiments, the production of rabbit antisera was carried out by YouLong Biotech.

### Enzyme-linked immunosorbent assay

One hundred nanogram of recombinant protein was dissolved in ELISA coating solution (Na_2_CO_3_ and NaHCO_3_, pH 9.6) and then coated with ELISA plate for overnight at 4°C. After the ELISA plate abandons the coated liquid, added 200 µL washing liquid (1 × TBST) to each well for three times, and then add 200 µL blocking solution (BSA in washing liquid) at room temperature for 2 h. The obtained immunized mouse serum was continuously diluted with ELISA diluent (BSA in washing liquid) (1:10,000 ~ 1:5,120,000). ELISA plates were washed with washing solution for three times, and 100 µL diluted serum was added to each well and placed in the chamber for 1.5 h. Goat anti-mouse HRP-IgG was diluted by ELISA diluent at 1:5,000 and was added into ELISA plates at room temperature for 1.5 h. After washing ELISA plate for three times, add 100 µL 1 × TMB solution to each well for color development at room temperature, and away from light for 3 min, ELISA stop solution 50 µL was added to each well immediately to stop the reaction for 2 min. After that, the OD450 value was read by enzyme label analyzer.

### Hematoxylin and eosin staining

Mouse spleen samples were fixed in 4% paraformaldehyde (Meilunbio, China) overnight. Fixed mouse spleens were placed in the embedding box and rinsed with running water for 1 h. Buried box successively were dehydrated in 70%, 85%, and 95% ethanol for 60 min and then transferred to anhydrous ethanol for 30 min twice. The embedding box was transferred to xylene and soaked twice for 15 min each time. The treated embedding box was immersed in melted paraffin wax for three times. Then, adjusted the position of the tissue in the embedding box and placed it in the mold. Carefully added melted paraffin and placed it in the cooling table. Section thickness was set to 4 µm, and the cooled paraffin tissue blocks were fixed and then sliced. The complete slices were put into the bleach machine to stretch them, and the slides were picked up and placed in the oven to dry. Dewaxed the slices twice in xylene, and the slices were soaked in anhydrous ethanol, 90% ethanol, 70% ethanol, and then distilled wash in water, ddH_2_O was rinsed after hematoxylin staining. The sections were soaked in 1% hydrochloric alcohol and rinsed with ddH_2_O until the sections turned blue. Then, eosin was dyed for 1 min and rinsed with ddH_2_O. Dehydrated in anhydrous ethanol, 95%, 75%, and 50% gradient ethanol, and then transparent in xylene. The neutral gum was sealed and observed under microscope.

### SDS-PAGE and western blot

Purified recombinant proteins were mixed with 5 × SDS reducing loading buffer and boiled for 8 min at 100°C and then resolved on 10%–12% SDS-PAGE followed by Coomassie brilliant blue staining (Beyotime) . For Western blot analysis, Proteins were sampled in 25 µg volumes. The proteins were separated by SDS-PAGE and transferred onto polyvinylidene difluoride membranes (Immobilon). Membranes were blocked with 5% skim milk in Tris-buffered saline containing Tween-20 (TBST) for 2 h at room temperature, the polyvinylidene difluoride membranes were incubated with primary antibodies overnight at 4°C and then with a horseradish peroxidase-labeled goat anti-mouse or anti-rabbit IgG antibody (SouthernBiotech) for 1.5 h at RT. After washing the membrane thrice with TBST, enhanced chemiluminescence (ECL, NCM biotech, Suzhou, China) was used to visualize the bands. The experiment was repeated three times, and the chemiluminescent signal was detected by the gel-imaging analysis system (Bio-Rad ChemiDoc MP) and analyzed by Image J software (Bio-Rad ChemiDoc). The following primary antibodies were used: mouse anti-His antibody (ABclonal), rabbit anti-GST antibody (1:2,000, Proteintech), rabbit anti-Flag antibody (Proteintech), rabbit anti-p65 antibody (1:1,000, Cell Signaling Technology), rabbit anti-p-p65 antibody (1:1,000, Cell Signaling Technology), rabbit anti-p38 antibody (1:1,000, Cell Signaling Technology), rabbit anti-p-p38 antibody (1:1,000, Cell Signaling Technology), rabbit anti-ERK antibody (1:1,000, Cell Signaling Technology), rabbit anti-p-ERK antibody (1:1,000, Cell Signaling Technology), rabbit anti-PySRA-F1/F2/F3 antisera, and rabbit anti-CD68 antisera (YouLong Biotech).

### Co-affinity purification and LC-MS analysis

Purified His-PySRA-F2 tag protein was used for the co-affinity purification of binding proteins from the RAW264.7 membrane. The RAW264.7 membrane protein was prepared according to cell membrane protein extraction kit (Thermo Fisher Scientific). His-PySRA-F2 protein was incubated with 500 µL equilibrated HisPur Ni-NTA Resin (QIAGEN) for 3 h at 4°C with rotation, followed by washing three times with 200 µL wash buffer (50 mM NaH_2_PO_4_, 300 mM NaCl, 20 mM imidazole, pH 8.0). RAW264.7 membrane proteins (500 µg) were added and incubated with the resin bound to His-PySRA-F2 protein overnight at 4°C. After washing the resin with 500 µL wash buffer to remove the unbound proteins, the bound complexes were eluted using 200 µL elution buffer (50 mM NaH_2_PO_4_, 300 mM NaCl, 200 mM imidazole, pH 8.0). The eluted proteins and purified His-tagged proteins were mixed with SDS-reducing loading buffer, boiled, and loaded on SDS-PAGE. The gels were then stained using the fast silver stain kit (Beyotime Biotechnology).

The differential protein bands were excised from the gels and digested using in-gel tryptic digestion. Remaining peptides were extracted from the gel with 60% acetonitrile containing 0.1% trifluoroacetic acid followed by ultrasonic treatment. The extracts were combined, dried in a vacuum concentrator, and dissolved in 0.1% formic acid for LC-MS analysis with a Q-Exactive mass spectrometer (Thermo Fisher Scientific) coupled to an Easy-nLC 1000 instrument (Thermo Fisher Scientific). Identification of protein results through the database (uniprot_Mus_musculus_88068_20210106).

### Pull-down assay

To evaluate the interaction between CD68 and PySRA-F2, His-pull-down assays were carried out. PySRA-F2 protein was incubated with anti-His beads (Sigma) for 3 h at 4°C to immobilize on beads. The beads were collected using a magnetic separator and incubated separately with 600 µg purified GST, GST-CD68, and GST-CKAP4 proteins for 12 h at 4°C. After washing the beads with 20 volumes of TBS buffer (50 mM Tris HCl, 150 mM NaCl, pH 7.4) three times, the beads were resuspended in 50 µL of SDSreducing loading buffer and boiled for 8 min. The supernatant was used for Western blot.

### Flow cytometry

When macrophage RAW264.7 was in the logarithmic growth stage, the culture medium was abandoned and washed with PBS. After that, the cells were slowly blown down with fresh culture solution and counted by cell-counting plate. 500,000 cells were placed in a sterile EP tube and the experimental group (RAW264.7 + 50 µg PySRA-F1 recombinant protein, RAW264.7 + 50 µg PySRA-F2 recombinant protein, RAW264.7 + 50 µg PySRA-F3 recombinant protein) and control group (RAW264.7), the culture medium was supplemented to 500 µL total volume, each group was set three multiple holes. The EP tube was placed on the silent mixer and rotated at room temperature for 3 h, centrifuged at 2,000 rpm at 4°C for 5 min, and the supernatant was discarded. Add 500 µL pre-cooled PBS and wash twice. Alexa Fluor 647 anti-His Tag Antibody diluted with PBS at 1:200. Then, added 50 µL to each tube. After standing on ice for 20 min and dyeing away from light, precooled PBS was washed three times at 2,000 rpm at 4°C. After centrifugation for 5 min, the supernatant was discarded, and 400 µL PBS was added for resuspension. The fluorescence intensity was detected by flow cytometry (BD FACSAria III).

For the detection of macrophage apoptosis by flow cytometry, when macrophage RAW264.7 was in logarithmic growth stage, the culture medium was abandoned and washed with PBS. After that, the cells were slowly blown down with fresh culture solution and counted by cell counting plate. 650,000 cells were placed in 12-well plates. DMEM complete culture solution containing 10 µg/mL PySRA-F2 recombinant protein was filtered through a 0.22-µm filter and then added 1 mL to 12-well plates. After treatment, washed twice with PBS and resuspended in 200 µL PBS. For apoptosis analysis, Annexin V-FITC/PI Apoptosis Detection Kit (Yeasen Biotechnology, Shanghai) was utilized according to the manufacturer’s instructions. Five microliters of Annexin V-FITC and 10 μL PI Staining Solution were added to each well, and the cells were incubated in the dark for 15 min. The fluorescence intensity was detected by flow cytometry (BD FACSAria III).

### Cloning, expression, and purification of recombinant CKAP4 proteins and CD68 proteins

The ckap4 (aa 1,336–1,668) and cd68 (aa 417–873) containing a Flag-tag at the C terminal were amplified from the cDNA (reversely transcribed by the RAW264.7 mRNA) using two respective reverse primers which introduced the HA tag nucleotide sequence and two respective forward primers listed in Table S1. PCR products were cloned into the BamHI and XhoI sites of pGEX-6P-1 vector. The pGEX-6P-1 plasmids carrying ckap4 and cd68 gene fragments were transformed into *E. coli* BL21 (DE3) pLysS cells for expression. Protein expression was induced with 0.5 mM IPTG for 7 h at 37°C.

### qRT-PCR

RAW264.7 cells (6.5 × 10^5^ cell/well) in a 12-well plate containing 10 µg/mL DMEM complete culture medium of PySRA-F2 recombinant protein was added into the pore and incubated with RAW264.7 for 12 h. Discard the medium and wash twice with pre-cooled PBS. RNA was extracted by qRT-PCR kit (Vazyme). Reverse transcription of the extracted RNA into cDNA (Yeasen) at 25°C for 5 min, 55°C for 15 min, and 85°C for 5 min (Primer sequences in Table S1).

### *P. yoelii* purification

When the protozoa rate reached about 50%, mouse eyeball blood was collected to contain 200 µL anticoagulant, mixed in EP tubes, and centrifuged at 2,000 rpm for 10 min at 4°C. Discarded supernatant and added RPMI 1640 for culture fluid resuspension cells. After rinsing NFW column with RPMI 1640 culture solution, the suspended cell suspension was passed through the column to remove white blood cells. After centrifugation at 2,000 rpm for 10 min, the supernatant was discarded and 7 mL RPMI 1640 culture solution was added to resuspend the cells. The centrifuge was slowly increased to 72% percoll, centrifuged at 300 rcf at 20°C for 30 min, and the centrifuge speed was set to 0. According to the culture medium and the different densities of Plasmodium in each period, it was divided into four layers, from bottom to top, they were ring stage, percoll, trophozoite and schizont phase, and 1640 medium. Mouse serum immunized with PySRA recombinant protein (1:1,000) as primary antibody to recognize the natural protein, sheep anti-mouse HRP-IgG was used as the secondary antibody (ABclonal).

### Gene knockout

Mouse tail blood was collected in 1% saponin, voracylated for 1 min, centrifuged at 13,000 rpm for 5 min, and washed three times in PBS; then, the supernatant was discarded and added with ddH_2_O. The samples were cooked in a metal bath at 95°C for 10 min, and the supernatant was stored by centrifugation and as PCR amplification template. Search the sequences before and after *pysra* and *pysra*-F2 through the PlasmoDB database. The left and right homologous arms are 400–600 bp before and after the design. Designed the sgRNA sequence through the website (http://zifit.partners.org/ZiFiT/ CSquare9Nuclease.aspx). Five microliters of purified schizonite phase cells and 5 µg knockout plasmids were constructed in a sterile EP tube, filled to 100 µL with electro-control converter, mixed gently, and transferred to the shock cup. Transfected with Lonza 2b electroscope T-16 program advance, resuspended with 200 µL PBS, and transferred into a 1 mL injection tube. Then, ICR mice (Cavens) were injected intravenously. After 24–36 h of electric transfer, tail blood smears of mice were taken and stained by Jimsa whether *plasmodium* infection is established in mice. When the results were positive, the water bottle was changed to contain 6 µg/mL pyrimethamine.

### Statistical analysis

All experimental data were expressed as mean ± standard deviation and analyzed by Microsoft Excel and GraphPad Prism5. One-way ANOVA was used for the comparison of multiple groups of samples, and then the SNK test was used for pound-for-pair comparison. Student *t* test was used to compare the two groups of independent samples, and the difference was statistically significant when *P* < 0.05.

## SUPPLEMENTAL MATERIAL

Fig. S1 iai.00113-24-s0001.tif Analysis of the impact of PvSRA on the host.

Fig. S2 iai.00113-24-s0002.tif HE staining results of mouse spleen infected with *P.y* 17XL.

Fig. S3 iai.00113-24-s0003.tif Construction of *pysra* knockout plasmid.

Supplemental legends iai.00113-24-s0004.docx Legends for Fig. S1 to S3.

Table S1 iai.00113-24-s0005.docx Amplified primer information.

ASM does not own the copyrights to Supplemental Material that may be linked to, or accessed through, an article. The authors have granted ASM a non-exclusive, world-wide license to publish the Supplemental Material files. Please contact the corresponding author directly for reuse.

## References

[1] WHO. 2022. World malaria report (2022). World Health Organization, Geneva.

[2] Gowda DC, Wu X. 2018. Parasite recognition and signaling mechanisms in innate immune responses to malaria. Front Immunol 9:3006. doi:10.3389/fimmu.2018.03006 30619355 PMC6305727

[3] Autino B, Corbett Y, Castelli F, Taramelli D. 2012. Pathogenesis of malaria in tissues and blood. Mediterr J Hematol Infect Dis 4:e2012061. doi:10.4084/MJHID.2012.061 23170190 PMC3499994

[4] Anstey NM, Russell B, Yeo TW, Price RN. 2009. The pathophysiology of vivax malaria. Trends Parasitol 25:220–227. doi:10.1016/j.pt.2009.02.003 19349210

[5] Miller LH, Baruch DI, Marsh K, Doumbo OK. 2002. The pathogenic basis of malaria. Nature 415:673–679. doi:10.1038/415673a 11832955

[6] Moxon CA, Gibbins MP, McGuinness D, Milner DA, Jr., Marti M. 2020. New insights into malaria pathogenesis. Annu Rev Pathol 15:315–343. doi:10.1146/annurev-pathmechdis-012419-032640 31648610

[7] Popa GL, Popa MI. 2021. Recent advances in understanding the inflammatory response in malaria: a review of the dual role of cytokines. J Immunol Res 2021:7785180. doi:10.1155/2021/7785180 34790829 PMC8592744

[8] Stevenson MM, Riley EM. 2004. Innate immunity to malaria. Nat Rev Immunol 4:169–180. doi:10.1038/nri1311 15039754

[9] Schofield L, Grau GE. 2005. Immunological processes in malaria pathogenesis. Nat Rev Immunol 5:722–735. doi:10.1038/nri1686 16138104

[10] Miller LH, Ackerman HC, Su XZ, Wellems TE. 2013. Malaria biology and disease pathogenesis: insights for new treatments. Nat Med 19:156–167. doi:10.1038/nm.3073 23389616 PMC4783790

[11] Gazzinelli RT, Kalantari P, Fitzgerald KA, Golenbock DT. 2014. Innate sensing of malaria parasites. Nat Rev Immunol 14:744–757. doi:10.1038/nri3742 25324127

[12] Chang KH, Stevenson MM. 2004. Malarial anaemia: mechanisms and implications of insufficient erythropoiesis during blood-stage malaria. Int J Parasitol 34:1501–1516. doi:10.1016/j.ijpara.2004.10.008 15582527

[13] Awandare GA, Kempaiah P, Ochiel DO, Piazza P, Keller CC, Perkins DJ. 2011. Mechanisms of erythropoiesis inhibition by malarial pigment and malaria-induced proinflammatory mediators in an in vitro model. Am J Hematol 86:155–162. doi:10.1002/ajh.21933 21264897 PMC4703402

[14] Dunst J, Kamena F, Matuschewski K. 2017. Cytokines and chemokines in cerebral malaria pathogenesis. Front Cell Infect Microbiol 7:324. doi:10.3389/fcimb.2017.00324 28775960 PMC5517394

[15] Götz A, Tang MS, Ty MC, Arama C, Ongoiba A, Doumtabe D, Traore B, Crompton PD, Loke P, Rodriguez A. 2017. Atypical activation of dendritic cells by Plasmodium falciparum. Proc Natl Acad Sci USA 114:E10568–E10577. doi:10.1073/pnas.1708383114 29162686 PMC5724257

[16] Clark IA, Alleva LM, Budd AC, Cowden WB. 2008. Understanding the role of inflammatory cytokines in malaria and related diseases. Travel Med Infect Dis 6:67–81. doi:10.1016/j.tmaid.2007.07.002 18342278

[17] Buffet PA, Safeukui I, Deplaine G, Brousse V, Prendki V, Thellier M, Turner GD, Mercereau-Puijalon O. 2011. The pathogenesis of Plasmodium falciparum malaria in humans: insights from splenic physiology. Blood 117:381–392. doi:10.1182/blood-2010-04-202911 20852127 PMC3031473

[18] Del Portillo HA, Ferrer M, Brugat T, Martin-Jaular L, Langhorne J, Lacerda MVG. 2012. The role of the spleen in malaria. Cell Microbiol 14:343–355. doi:10.1111/j.1462-5822.2011.01741.x 22188297

[19] Chua CL, Brown G, Hamilton JA, Rogerson S, Boeuf P. 2013. Monocytes and macrophages in malaria: protection or pathology? Trends Parasitol 29:26–34. doi:10.1016/j.pt.2012.10.002 23142189

[20] Amlabu E, Mensah-Brown H, Nyarko PB, Akuh OA, Opoku G, Ilani P, Oyagbenro R, Asiedu K, Aniweh Y, Awandare GA. 2018. Functional characterization of Plasmodium falciparum surface-related antigen as a potential blood-stage vaccine target. J Infect Dis 218:778–790. doi:10.1093/infdis/jiy222 29912472 PMC6057521

[21] Yang B, Liu H, Xu QW, Sun YF, Xu S, Zhang H, Tang JX, Zhu GD, Liu YB, Cao J, Cheng Y. 2021. Genetic diversity analysis of surface-related antigen (SRA) in Plasmodium falciparum imported from Africa to China. Front Genet 12:688606. doi:10.3389/fgene.2021.688606 34421996 PMC8378275

[22] Yu JL, Liu QY, Yang B, Sun YF, Wang YJ, Jiang J, Wang B, Cheng Y, Wang QB. 2022. Immunogenicity analysis of the recombinant Plasmodium falciparum surface-related antigen in mice. Pathogens 11. doi:10.3390/pathogens11050550 PMC9145071 35631071

[23] Fu H, Lu J, Zhang X, Wang B, Sun Y, Lei Y, Shen F, Kassegne K, Han ET, Cheng Y. 2021. Identification of the recombinant Plasmodium vivax surface-related antigen as a possible immune evasion factor against human splenic fibroblasts by targeting ITGB1. Front Cell Dev Biol 9:764109. doi:10.3389/fcell.2021.764109 34938733 PMC8685506

[24] Clark IA, Budd AC, Alleva LM, Cowden WB. 2006. Human malarial disease: a consequence of inflammatory cytokine release. Malar J 5:85. doi:10.1186/1475-2875-5-85 17029647 PMC1629020

[25] Henry B, Roussel C, Carucci M, Brousse V, Ndour PA, Buffet P. 2020. The human spleen in malaria: filter or shelter?. Trends Parasitol 36:435–446. doi:10.1016/j.pt.2020.03.001 32298631

[26] Perry JA, Rush A, Wilson RJ, Olver CS, Avery AC. 2004. Dendritic cells from malaria-infected mice are fully functional APC. J Immunol 172:475–482. doi:10.4049/jimmunol.172.1.475 14688357

[27] Engwerda CR, Beattie L, Amante FH. 2005. The importance of the spleen in malaria. Trends Parasitol 21:75–80. doi:10.1016/j.pt.2004.11.008 15664530

[28] Looareesuwan S, Suntharasamai P, Webster HK, Ho M. 1993. Malaria in splenectomized patients: report of four cases and review. Clin Infect Dis 16:361–366. doi:10.1093/clind/16.3.361 8452947

[29] Chotivanich K, Udomsangpetch R, McGready R, Proux S, Newton P, Pukrittayakamee S, Looareesuwan S, White NJ. 2002. Central role of the spleen in malaria parasite clearance. J Infect Dis 185:1538–1541. doi:10.1086/340213 11992295

[30] Zhang HW, Li SJ, Hu T, Yu YM, Yang CY, Zhou RM, Liu Y, Tang J, Wang JJ, Wang XY, Sun YX, Feng ZC, Xu BL. 2017. Prolonged parasite clearance in a Chinese splenectomized patient with falciparum malaria imported from Nigeria. Infect Dis Poverty 6:44. doi:10.1186/s40249-017-0259-5 28372588 PMC5379605

[31] Urban BC, Ing R, Stevenson MM. 2005. Early interactions between blood-stage plasmodium parasites and the immune system. Curr Top Microbiol Immunol 297:25–70. doi:10.1007/3-540-29967-x_2 16265902

[32] Walther M, Woodruff J, Edele F, Jeffries D, Tongren JE, King E, Andrews L, Bejon P, Gilbert SC, De Souza JB, Sinden R, Hill AVS, Riley EM. 2006. Innate immune responses to human malaria: heterogeneous cytokine responses to blood-stage Plasmodium falciparum correlate with parasitological and clinical outcomes. J Immunol 177:5736–5745. doi:10.4049/jimmunol.177.8.5736 17015763

[33] Gilmore TD. 2006. Introduction to NF-kappaB: players, pathways, perspectives. Oncogene 25:6680–6684. doi:10.1038/sj.onc.1209954 17072321

[34] Kishore A, Petrek M. 2021. Roles of macrophage polarization and macrophage-derived miRNAs in pulmonary fibrosis. Front Immunol 12:678457. doi:10.3389/fimmu.2021.678457 34489932 PMC8417529

[35] Cha SJ, Park K, Srinivasan P, Schindler CW, van Rooijen N, Stins M, Jacobs-Lorena M. 2015. CD68 acts as a major gateway for malaria sporozoite liver infection. J Exp Med 212:1391–1403. doi:10.1084/jem.20110575 26216124 PMC4548058

[36] Holness CL, da Silva RP, Fawcett J, Gordon S, Simmons DL. 1993. Macrosialin, a mouse macrophage-restricted glycoprotein, is a member of the lamp/lgp family. J Biol Chem 268:9661–9666. 8486654

[37] Ishizaki T, Hernandez S, Paoletta MS, Sanderson T, Bushell ESC. 2022. CRISPR/Cas9 and genetic screens in malaria parasites: small genomes, big impact. Biochem Soc Trans 50:1069–1079. doi:10.1042/BST20210281 35621119 PMC9246331

[38] Hsu PD, Lander ES, Zhang F. 2014. Development and applications of CRISPR-Cas9 for genome engineering. Cell 157:1262–1278. doi:10.1016/j.cell.2014.05.010 24906146 PMC4343198

[39] Jinek M, Chylinski K, Fonfara I, Hauer M, Doudna JA, Charpentier E. 2012. A programmable dual-RNA-guided DNA endonuclease in adaptive bacterial immunity. Science 337:816–821. doi:10.1126/science.1225829 22745249 PMC6286148

