# Supplementary figures and images for: Plasmodium yoelii surface-related antigen (PySRA) modulates the host pro-inflammatory responses via binding to CD68 on macrophage membrane
Source: Infect Immun. 2024 Apr 16;92(5):e00113-24. doi: 10.1128/iai.00113-24 (PMC11075460; doi:10.1128/iai.00113-24)

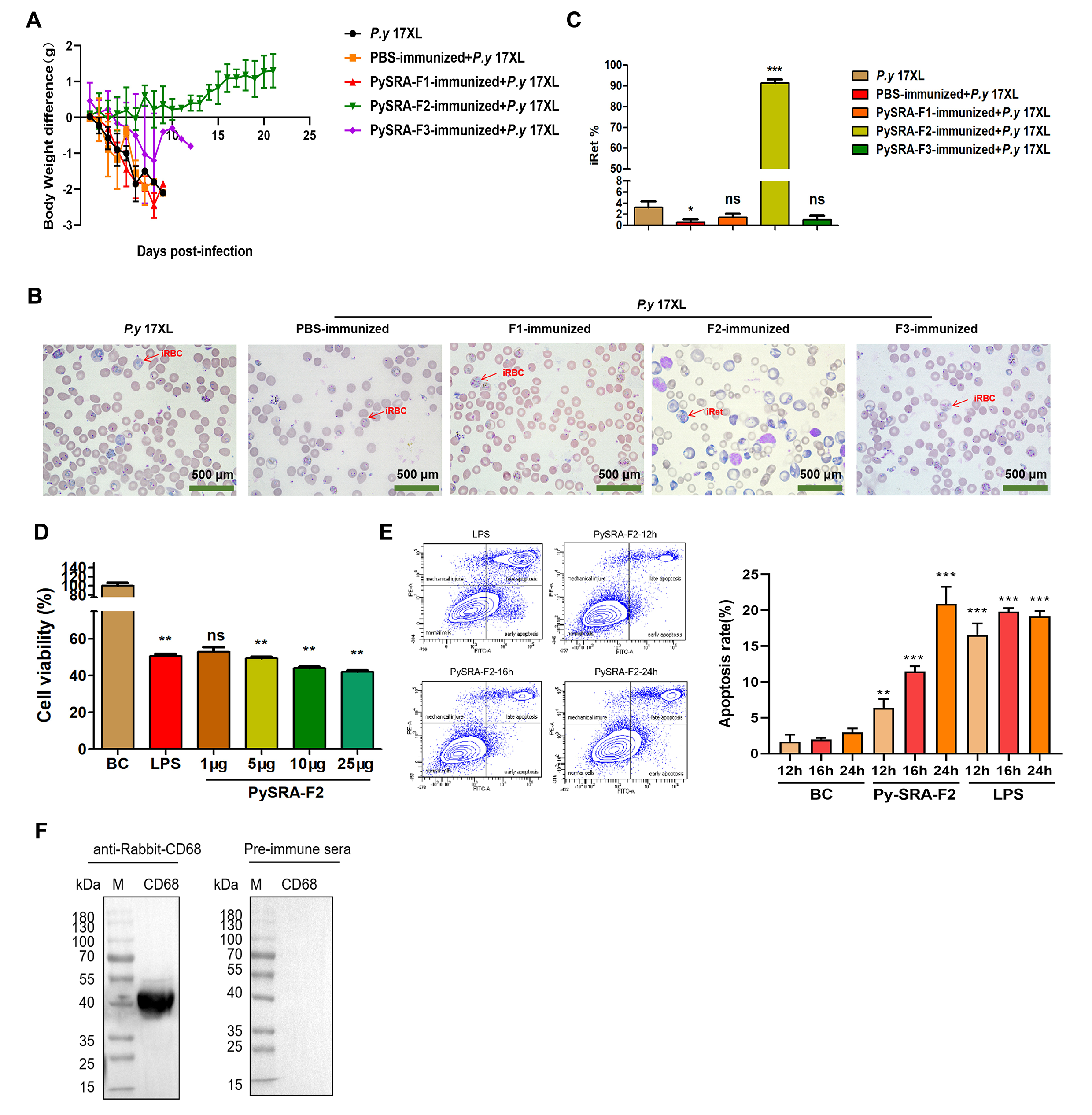

Supplement: Fig. S1 — Analysis of the impact of PvSRA on the host. [file iai.00113-24-s0001.tif]

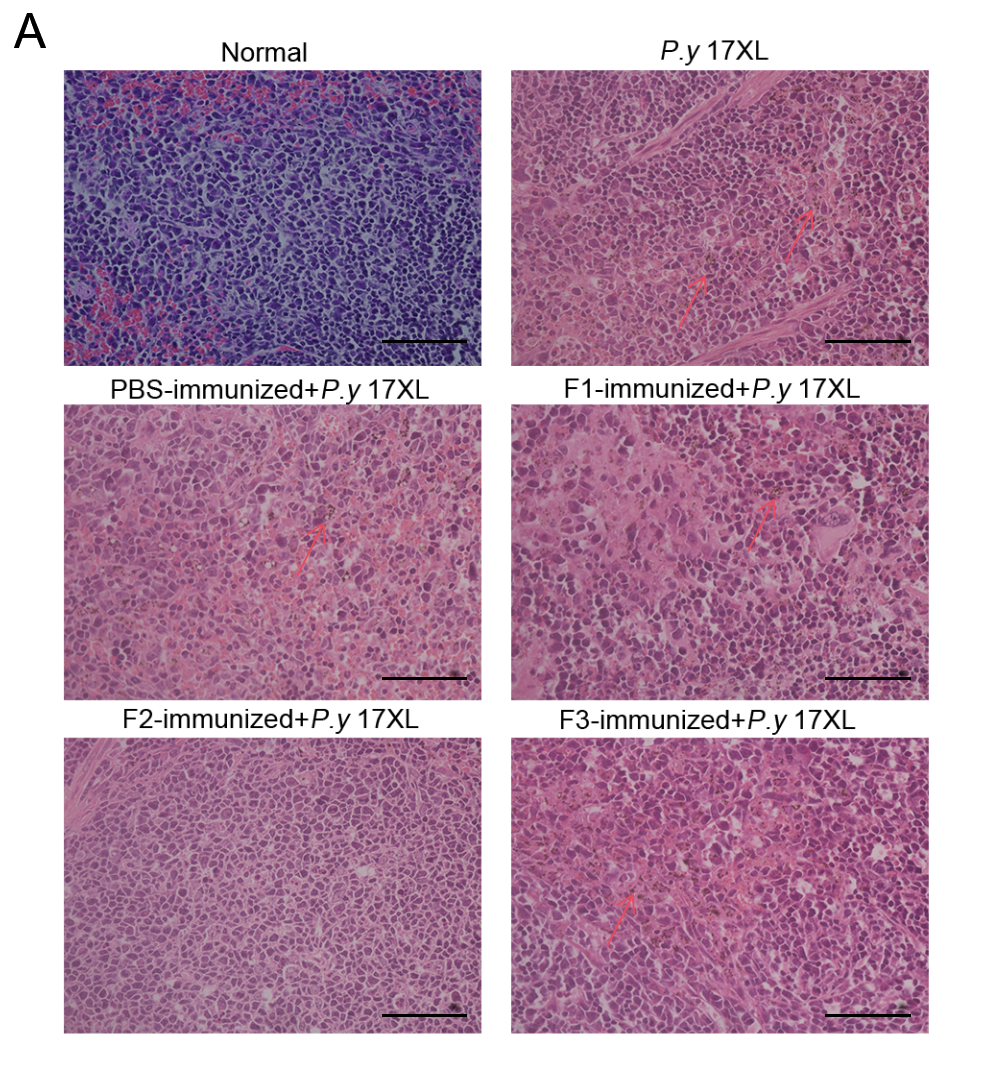

Supplement: Fig. S2 — HE staining results of mouse spleen infected with P.y 17XL. [file iai.00113-24-s0002.tif]

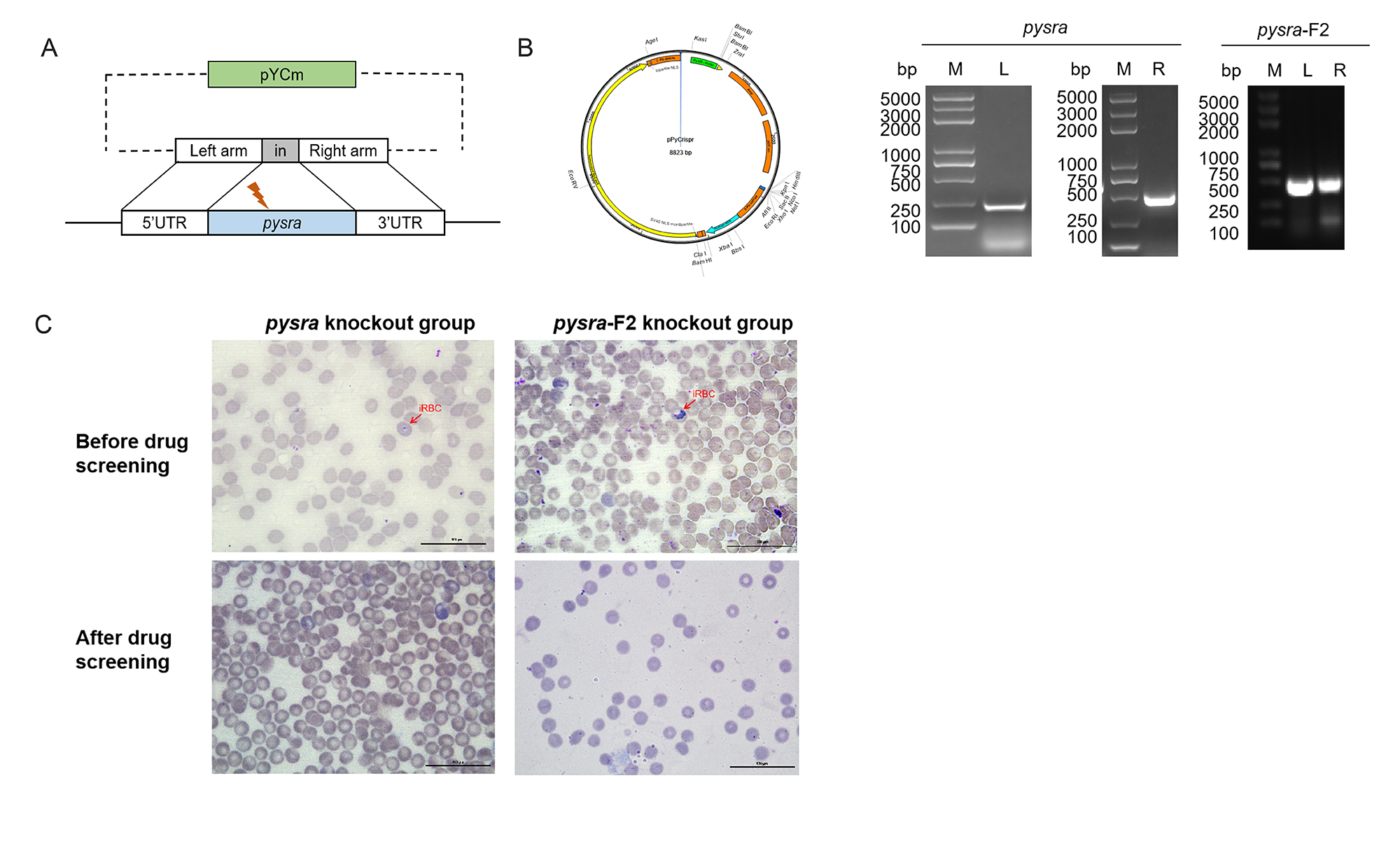

Supplement: Fig. S3 — Construction of pysra knockout plasmid. [file iai.00113-24-s0003.tif]
