# Supplementary material for: Plasmodium yoelii surface-related antigen (PySRA) modulates the host pro-inflammatory responses via binding to CD68 on macrophage membrane
Source: Infect Immun. 2024 Apr 16;92(5):e00113-24. doi: 10.1128/iai.00113-24 (PMC11075460; doi:10.1128/iai.00113-24)
Supplement: Supplemental legends — Legends for Fig. S1 to S3. [file iai.00113-24-s0004.docx]

**Supplementary Materials Figure S1.** Analysis of the impact of PvSRA on the host. (A) The weight change of immunized mice infected with *P.y* 17XL. (B) The blood smear of immunized mice infected with *P.y* 17XL. (C) The proportion of *Plasmodium* invasion to reticulocytes in 10 visual fields was statistically analyzed (iRet% = the number of *plasmodium* invasion reticulocytes/the number of plasmodium invasion erythrocytes) (**P* < 0.05; ****P* < 0.001; ns: no significance). (D) The activity of RAW264.7 cells were inhibited by PySRA-F2 (***P* < 0.01; ns: no significance). (E) PySRA-F2 induced apoptosis of RAW264.7 cells (***P* < 0.01; ****P* < 0.001; ns: no significance). (F) The polyclonal antibody specifically recognized CD68 protein. CD68 recombinant protein was recognized by polyclonal rabbit of anti-CD68 and the rabbit sera before immunization. One-way ANOVA was used for the comparison of multiple groups of samples, and then SNK test was used for pound-for-pair comparison. Student *t* test was used to compare the two groups of independent samples.

**Supplementary Materials Figure S2.** HE staining results of mice spleen infected with *P.y* 17XL. The red arrow indicates malarial hemozoin (Scale bars: 20 μm).

**Supplementary Materials Figure S3.** Construction of *pysra* knockout plasmid. (A) Schematic diagram of *pysra* knockout plasmid. (B) PCR amplification of homologous arms. L: Left homologous arm. R: Right homologous arm. M: DNA marker. (C) Blood smear results after pyrimethamine screening. *pysra* full-length and *pysra*-F2 gene knockout group before drug screening, after drug screening.
