# Supplementary material for: Plasmodium yoelii surface-related antigen (PySRA) modulates the host pro-inflammatory responses via binding to CD68 on macrophage membrane
Source: Infect Immun. 2024 Apr 16;92(5):e00113-24. doi: 10.1128/iai.00113-24 (PMC11075460; doi:10.1128/iai.00113-24)
Supplement: Table S1 — Amplified primer information. [file iai.00113-24-s0005.docx]

Table S1 The amplified primer information

| No. | Primer | Sequences (5'→3')^a^ |
| --- | --- | --- |
| 1 | pET32a-PySRA-F1-F | gctgatatcGGATCCAAACAGATCCAAAAGACCCTT |
| 2 | pET32a-PySRA-F1-R | gtggtggtgCTCGAGCTTATCGTCGTCATCCTTGTAATCGACCTGGTCATGCTTTCC |
| 3 | pET32a-PySRA-F2-F | gctgatatcGGATCCACTCAATTTTATGTCAATGACTATTC |
| 4 | pET32a-PySRA-F2-R | gtggtggtgCTCGAGCTTATCGTCGTCATCCTTGTAATCGCCGTTGCGCGACTT |
| 5 | pET32a-PySRA-F3-F | gctgatatcGGATCCATTGTCAAAAGTTGCAAGAATTC |
| 6 | pET32a-PySRA-F3-R | gtggtggtgCTCGAGCTTATCATCATCGTCCTTGTAGTC |
| 7 | pGEX-6P-1-CKAP4-F | gggcccctgGGATCCAGCACAGTCAGGAGCCTGG |
| 8 | pGEX-6P-1-CKAP4-R | atgcggccgCTCGAGCTTATCGTCGTCATCCTTGTAATCGTCATCCAGCAAGCCCTT |
| 9 | pGEX-6P-1-CD68-F | gggcccctgGGATCCGTTCAGCTCCAAGCCCAA |
| 10 | pGEX-6P-1-CD68-R | atgcggccgCTCGAGCTTATCGTCGTCATCCTTGTAATCGGACTGGTCACGGTTGCA |
| 11 | IL-1β-F | GGTGTGTGACGTTCCCATTAGAC |
| 12 | IL-1β-R | CATGGAGAATATCACTTGTTGGTTGA |
| 13 | iNOS-F | GAGCTCGGGTTGAAGTGGTATG |
| 14 | iNOS-R | GAAACTATGGAGCACAGCCACAT |
| 15 | TNF-α-F | CAGGCGGTGCCTATGTCTC |
| 16 | TNF-α-R | CGATCACCCCGAAGTTCAGTAG |
| 17 | β-actin-F | AGTGTGACGTTGACATCCG |
| 18 | β-actin-R | GCAGCTCAGTAACAGTCCGC |

^a^The vector sequences are lowercase, and the restriction sites are underlined. FLAG tag sequences is highlighted in gray.
